# Editorial: Body image following cancer treatment

**DOI:** 10.3389/fpsyg.2022.1068977

**Published:** 2022-11-07

**Authors:** Simon Dunne, Margaret Fitch, Cherith Semple

**Affiliations:** ^1^School of Psychology, Faculty of Science and Health, Dublin City University, Dublin, Ireland; ^2^Lawrence S. Bloomberg Faculty of Nursing, University of Toronto, Toronto, ON, Canada; ^3^School of Nursing and Paramedic Science, Institute of Nursing and Health Research, Ulster University, Coleraine, United Kingdom

**Keywords:** body image (MeSH), cancer survivorship, psycho-oncology, patient—centered care, health related QOL

Significant medical advances in screening, prevention, and successful treatment in recent decades have increased the number of people living with and beyond cancer worldwide every year (Hulvat, 2020). Many of these people are recommended to receive aggressive treatments in order to prevent metastasis and improve survival (Shrestha et al., 2019). The aggressive nature of these treatments can often lead to significant changes to an individual's body. This includes, but is not limited to, changes to their appearance (e.g., surgical scars, removal of individual body parts, limb swelling, anorexia, and hair loss), sensory changes (e.g., pain, numbness, tingling, burning), functional changes (e.g., changes to speech, swallowing, hearing, eyesight, bowel/bladder incontinence), sexuality/fertility effects, weight gain or weight loss, loss of mobility, and the need to use of prosthetic devices (Fingeret, 2011). These changes to an individual's body resulting from cancer treatment can have a substantial impact on their everyday life, particularly in relation to their body image.

Body image is a multidimensional construct describing an individual's mental representation of their body, including their total concept of conscious and unconscious feelings, thoughts, and perceptions about their bodies, as well as their awareness of how others perceive them (Alebachew and Ashagrie, 2017). In the context of cancer treatment, it has become common to examine body image as it relates to body image disturbances and associated distress. For instance (Rhoten, 2016) has identified the following core attributes of body image disturbance in adults who have undergone cancer treatment: (1) dissatisfaction with a perceived change in appearance resulting from cancer treatment; (2) decline in function relating to some aspect of one's body; and (3) psychological distress regarding these changes.

Body image disturbance and distress are known to affect a range of psychosocial factors for individuals following cancer treatment. In particular, heightened concerns relating to a changed appearance following cancer treatment may lead some individuals to avoid contact with others altogether and become isolated (Fingeret et al., 2014; Fingeret and Teo, 2018). Changes in body image resulting from cancer and its treatment may also have deleterious effects on other aspects of a person's daily life, such as their experiences of sexuality and relationships (Pelusi, 2006; Sacerdoti et al., 2010; Faria et al., 2021). However, while some studies have shown a direct relationship between self-reported dissatisfaction with body image and emotional, functional, physical, and social wellbeing following cancer treatment, such findings have been inconsistent in the literature, particularly in relation to cancers such as head and neck cancer (Howren et al., 2013).

The potential complexity of the relationship between body image and wellbeing following cancer treatment has been underscored by a diverse literature which has examined how socio-demographic and clinical factors can influence this relationship. For instance, a recent systematic review of older breast cancer survivors found that older women, particularly those who are post-menopausal, may be less affected by changes to their appearance following breast cancer treatment (Davis et al., 2020). In addition to these variables, other socio-demographic and clinical factors which have been demonstrated to affect the relationship between body image and wellbeing following cancer treatment, include gender, marital status, income, education level, cancer stage, treatment type, and many other such variables (Rezaei et al., 2016; Albert et al., 2022). This demonstrates the need for a nuanced understanding of the complex inter-relationship between body image and clinical, demographic, functional, and psychosocial variables. However, there is still a limited literature base examining the impact of such relationships longitudinally.

The inherent subjectivity in the experience of body image following cancer treatment has also been noted in the literature. It is increasingly recognized that sensitivity toward bodily changes following cancer treatment varies among those affected and is not necessarily proportional to the amount of change experienced (Rhoten, 2016). A growing body of research has begun to examine the differential subjective impact of body changes following cancer treatment by investigating the mediating or moderating influence of particular variables on the relationship between body image and wellbeing. For instance, investment in appearance has been identified as a variable that may have a particularly important moderating effect on the relationship between body image and emotional wellbeing (Helms et al., 2008; Sherman et al., 2017). There is a need to continue this work of disambiguating the subjective experience of body image following cancer treatment through examining possible mediating and moderating variables, and through qualitative research examining the subjective experience of living with a changed body following cancer treatment.

The increasing use of body image screening and interventions in oncology settings also underscores the importance of this topic. Body image measurement and screening is particularly important to allow health professionals and intervention developers to identify at-risk individuals who may be targeted for tailored interventions that ameliorate the deleterious effects of cancer treatment on body image among cancer survivors. However, there is a growing recognition of the lack of gold standard approaches to body image screening and measurement (Covrig et al., 2021). In relation to body image interventions, systematic reviews have highlighted the potential usefulness of cognitive-behavioral therapy, social interaction skills training, and physical activity interventions in promoting positive body image (Lewis-Smith et al., 2018). Nevertheless, there are inherent methodological limitations and limited use of randomized controlled trials in this work, which make it difficult to draw definitive conclusions about the efficacy of such interventions (Lewis-Smith et al., 2018). There is also a need for more tailored interventions that focus on developing positive body image in relation to men or for cancer populations beyond breast cancer (Esplen and Fingeret, 2021).

The articles presented in the current collection go some way to addressing core issues in the literature base, which we have identified above. The collection brings together quantitative research exploring the longitudinal relationship between body image and wellbeing and mediating factors involved in this relationship, qualitative research, and patient-driven perspectives which spotlights cancer survivors' body image needs and intervention preferences following cancer treatment, and reviews of the literature and commentaries which synthesize important learnings from research on body image in specific cancer groups. We also present some exciting new work, involving the validation of novel scales addressing body image and formal evaluations of interventions designed to improve outcomes among cancer survivors who are impacted by body image changes.

## Author contributions

SD: conceptualization, original manuscript preparation, and writing—review and editing. MF and CS: conceptualization, manuscript review, and editing. All authors contributed to the article and approved the submitted version.

## Conflict of interest

The authors declare that the research was conducted in the absence of any commercial or financial relationships that could be construed as a potential conflict of interest.

## Publisher's note

All claims expressed in this article are solely those of the authors and do not necessarily represent those of their affiliated organizations, or those of the publisher, the editors and the reviewers. Any product that may be evaluated in this article, or claim that may be made by its manufacturer, is not guaranteed or endorsed by the publisher.
